# New ways of estimating excess mortality of chronic diseases from aggregated data: insights from the illness-death model

**DOI:** 10.1186/s12889-019-7201-7

**Published:** 2019-06-28

**Authors:** Ralph Brinks, Thaddäus Tönnies, Annika Hoyer

**Affiliations:** 1 Institute for Biometry and Epidemiology, German Diabetes Center, Auf’m Hennekamp 65, 40225 Duesseldorf, Germany; 2 Department and Hiller Research Unit for Rheumatology, University Hospital Duesseldorf, Moorenstr. 5, 40225 Duesseldorf, Germany

**Keywords:** Chronic disease epidemiology, Multi-state model, Prevalence, Incidence, Dementia, Diabetes, Partial differential equation, Bayes estimation

## Abstract

**Background:**

Recently, we have shown that the age-specific prevalence of a disease can be related to the transition rates in the illness-death model via a partial differential equation (PDE). The transition rates are the incidence rate, the remission rate and mortality rates from the ‘Healthy’ and ‘Ill’ states. In case of a chronic disease, we now demonstrate that the PDE can be used to estimate the excess mortality from age-specific prevalence and incidence data. For the prevalence and incidence, aggregated data are sufficient - no individual subject data are needed, which allows application of the methods in contexts of strong data protection or where data from individual subjects is not accessible.

**Methods:**

After developing novel estimators for the excess mortality derived from the PDE, we apply them to simulated data and compare the findings with the input values of the simulation aiming to evaluate the new approach. In a practical application to claims data from 35 million men insured by the German public health insurance funds, we estimate the population-wide excess mortality of men with diagnosed type 2 diabetes.

**Results:**

In the simulation study, we find that the estimation of the excess mortality is feasible from prevalence and incidence data if the prevalence is given at two points in time. The accuracy of the method decreases as the temporal difference between these two points in time increases. In our setting, the relative error was 5% and below if the temporal difference was three years or less. Application of the new method to the claims data yields plausible findings for the excess mortality of type 2 diabetes in German men.

**Conclusions:**

The described approach is useful to estimate the excess mortality of a chronic condition from aggregated age-specific incidence and prevalence data.

**Trial registration:**

The article does not report the results of any health care intervention.

**Electronic supplementary material:**

The online version of this article (10.1186/s12889-019-7201-7) contains supplementary material, which is available to authorized users.

## Background

**Fig. 1:**
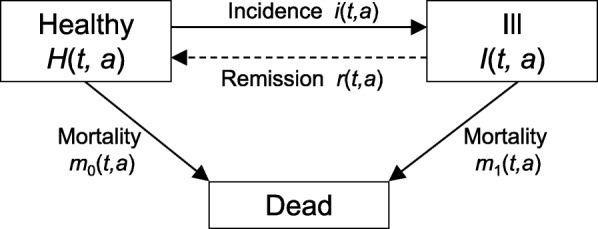
Illness-death model. The transition rates *i* (incidence), *r* (remission), *m*_0_ (mortality of the healthy), *m*_1_ (mortality of the diseased) between the compartments depend on calendar time *t* and age *a*. In case of chronic diseases, there is no way back from the *Ill* state to the *Healthy* state (dashed line). Then, the remission rate *r* equals zero

Recently, we have shown that the age-specific prevalence of a health state or disease can be related to the transition rates in the illness-death model via a partial differential equation (PDE) [1, 2]. The transition rates are the incidence rate, the remission rate and mortality rates from the *Healthy* and *Ill* states (Fig. 1). In case of a chronic disease, i.e. a disease with no remission, this relation can be used to estimate the incidence from a sequence of cross-sectional studies if information about mortality is available [3]. This might be an alternative way to estimate the incidence of a chronic condition in situations where follow-up studies are challenging to conduct or not feasible at all.

In this article, we demonstrate that it is also possible to estimate excess mortality from age-specific prevalence and incidence of a chronic disease. This can be useful for the analysis of data where it is difficult to observe mortality directly, for instance in disease registers [4] or health insurance claims data where cases of death might be reported with a delay [5]. Another example where excess mortality of a chronic condition cannot be estimated directly is the US National Health Interview Survey (NHIS) from the National Center for Health Statistics [6]. NHIS is a yearly cross-sectional household interview survey with up to 90,000 participants each year. Usually, participants are followed up for mortality by linkage to the National Death Index. This implies that it is possible to check the vital status of a participant from a previous cross-sectional interview, but it is not possible to decide if a deceased participant who had been disease-free at the interview, has contracted the disease in the period between the cross-section and the date of death. With other words, for a subject disease-free at the interview, it is not possible to determine the disease status at death. Thus, in estimating the mortality it is uncertain to attribute this case to the mortality of the healthy or of the diseased subjects.

To overcome these problems, we examine mathematical relations of the illness-death model and associated PDEs to develop reliable estimators for excess mortality.

## Methods

### Illness-death model

We consider the illness-death model as shown in Fig. 1. Each subject of the population is in one of the relevant disease states, *Healthy* (with respect to the considered chronic disease) *Ill* or *Dead*. Let the number of people aged *a* at calendar time *t* in the *Healthy* and *Ill* states be denoted by *H*(*t*, *a*) and *I*(*t*, *a*), respectively. Subjects can transit from both states into the (absorbing) state *Dead*. The transition rates between the three states are the incidence rate (*i*), the remission rate (*r*), the mortality rate of the healthy (*m*_0_) and the mortality rate of the diseased (*m*_1_). These rates usually depend on calendar time *t* and on age *a*. Henceforth, we consider only chronic, i.e., irreversible diseases, which is equivalent to a remission rate of zero (*r* = 0).

To develop estimators for the excess mortality Δ*m* = *m*_1_ – *m*_0_, we use mathematical relations between the incidence, prevalence *p*(*t*, *a*) = *I*(*t*, *a*)/{*I*(*t*, *a*) + *H*(*t*, *a*)} and the mortality rates in the illness-death model.

An alternative epidemiological measure to Δ*m* for assessing discrepancies between the mortality rates *m*_0_ and *m*_1_, is the mortality rate ratio *R* = *m*_1_/*m*_0_ which is of potential interest for practitioners. The mortality rate ratio *R* expresses the mortality rate of the diseased people *relative* to the non-diseased at the same age. Due to this plain interpretation, *R* is more often used than the (absolute) excess mortality Δ*m*. Both measures, Δ*m* and *R*, are related by *R* = 1 + Δ*m*/*m*_0_.

### Direct estimation in simulated data about dementia

**Fig. 2:**
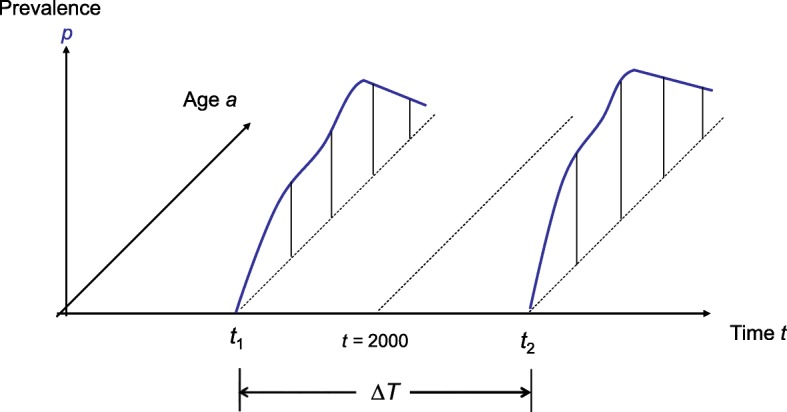
Prevalence data from two cross-sections at time *t*_1_ and *t*_2_ are used to estimate the excess mortality midpoint at *t* = *t*_1_ + ΔT/2 = *t*_2_ - ΔT/2 (figure adopted from [Bri16]])

To illustrate how measures of excess mortality in a chronic disease can directly be estimated from incidence and prevalence data, we conduct a simulation study. We mimic a sequence of two cross-sectional studies for a chronic disease in two different years *t*_1_ and *t*_2_ centered at the year *t* = 2000. Let Δ*T* = *t*_2_ – *t*_1_denote the difference between *t*_1_ and *t*_2_, i.e. *t*_1_ = 2000 – Δ*T*/2 and *t*_2_ = 2000 + Δ*T*/2. In each of the cross-sectional studies at *t*_1_ and *t*_2_, the age-specific prevalence *p* is surveyed (Fig. 2).

The aim is to estimate the excess mortality at year *t* = 2000 from the cross-sectional prevalence data at *t*_1_ and *t*_2_ and the incidence. To assess the impact of the temporal difference between the cross-sectional studies, we vary Δ*T* from 0.1 to 10 (years). Together with the age-specific incidence rate *i* at *t* = 2000, the prevalence data in the two years *t*_1_ and *t*_2_ serve as input values to estimate the excess-mortality in the year *t* = 2000. The estimated excess mortality is then compared with the rates used to set up the simulation study in terms of absolute and relative bias.

The input data for the simulation are motivated from survey data about dementia in the female population of Europe [7]. Dementia is a major health problem in many countries with potentially increasing prevalence in the future [8]. The age-specific prevalence *p* for each of the two years *t*_1_ and *t*_2_ is calculated analytically with the incidence rate *i* from [7]. The age-specific mortality rate *m*_0_ of the dementia-free population is chosen to be *m*_0_(*t*, *a*) = exp.(− 10.7 + 0.1*a* + *t* ln(0.99)) aiming to approximate the mortality of the European population based on the Gompertz-Makeham law of mortality [9]. In addition, we assume that the mortality *m*_1_ of the diseased people can be written as a product of *m*_0_ and *R*: *m*_1_(*t*, *a*) = *R*(*t*, *a*) × *m*_0_(*t*, *a*) with log *R*(*t*, *a*) = log(3) + [log(1.5) – log(3)] (*a* – 60)/(90–60). The rationale for choosing this *R* is based on the idea that *m*_1_ also follows a Gompertz-Makeham law. Then, the logarithm of the quotient *m*_1_/*m*_0_ is a straight line as given here. The specific numerical values in the definition of *R* are chosen to mimic the age-dependency as reported in [10], where *R* was found to be about 3 and 1.5 at 60 and 90 years of age, respectively. Note, however, that in this simulation we want to demonstrate feasibility of the method in a realistic range of parameters. We do not aim for the best obtainable agreement between our input data and the observed data.

### Bayes estimation and application to claims data

**Fig. 3:**
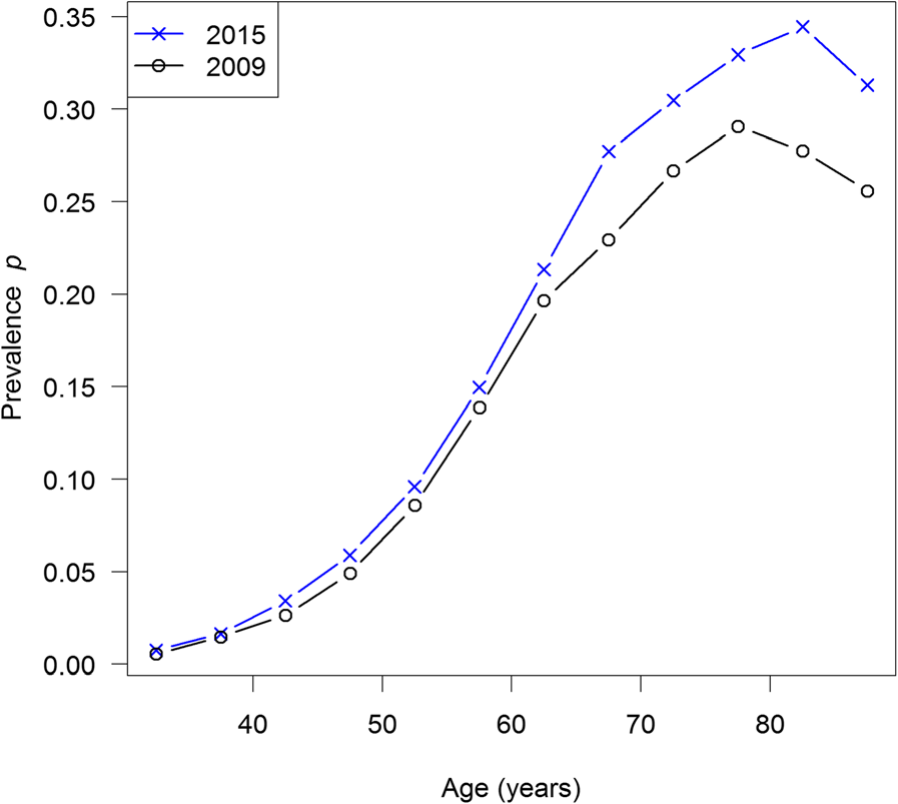
Surveyed age-specific prevalence *p* of type 2 diabetes in German men in 2009 (black line with circles) and 2015 (blue with crosses)

After describing the direct estimation, we present an estimation method in the framework of Bayesian inference. Bayes methods are increasingly used in applied statistics because they provide a flexible framework for the analysis of scientific problems and quantifying uncertainty in their solution [11]. As an application of the Bayesian approach, we estimate the excess mortality of type 2 diabetes in the year 2012 from claims data comprising 35 million German men. Goffrier and colleagues [12] reported the age-specific prevalence of diabetes among German men in the years *t*_1_ = 2009 and *t*_2_ = 2015 as shown in Fig. 3. In the same work, the age-specific incidence rate *i* in 2012 has been surveyed. The data for this analysis is publicly available and can be found in [12].

Our aim in the diabetes example is to estimate the age-specific mortality rate ratio *R* in the range 50 to 90 years of age. Recently, for a smaller age range the mortality rate ratio has been estimated in Tönnies et al. [13]. Compared to [13] we extend the age range by the novel Bayesian approach.

The idea for the Bayes method is that for given age-specific prevalence *p*, incidence rate *i* and general mortality *m*, an estimate of the excess mortality in terms of the mortality rate ratio *R* is desired. According to the Theorem of Bayes [11] we obtain.

1 $$ \mathrm{f}\left(R\ |p\right)\propto \mathrm{f}\left(p|R\right)\ \mathrm{x}\ \mathrm{f}(R) $$

where f(*R* |*p*) is the posteriori distribution of *R*, f(*p*|*R*) is the probability density function of *p* given *R* and f(*R*) denotes the priori distribution of *R*. For clarity, we assume that *i* and *m* are known. Motivated by empirical findings from the Danish Diabetes Register [14], we assume that the logarithm of the age-specific mortality rate ratio *R* approximately is a straight line in the age range 50 to 90 years:

2 $$ \log \left(R(a)\right)=\log \left(R(50)\right)+\left[\log \left(R(90)\right)-\log \left(R(50)\right)\right]\ \left(a-50\right)/\left(90-50\right) $$

For estimation of *R*(50) and *R*(90) in Eq. (2), we use weakly informative prior distributions *R*(50) ~ U(2; 9) and *R*(90) ~ U(1; 2); again inspired by the Danish diabetes register. U(*v*; *w*) means the continuous uniform distribution with minimum and maximum value *v* and *w*, respectively. In Bayesian terminology, our aim is to estimate the joint a-posteriori distribution for *R*(50) and *R*(90).

To use Eq. (1) for the estimation of *R* given *p*, we apply three steps: 1) values for *R*(50) and *R*(90) are drawn from the uniform prior distributions, 2) solving the PDE with initial condition *p*(2009; *a*) as given in [12] and 3) comparing the calculated solution *p* in 2015 with the surveyed values.

For solving the PDE, we use the Method of Characteristics [15] to first convert the PDE into an ordinary differential equation (ODE) and then, second, solve the ODE by the Runge-Kutta Method of fourth order [16]. Next, the calculated prevalence in 2015, *p*(2015; *a*), is compared with the observed prevalence in 2015 given by [12]. The age-specific prevalences *p* in the years 2009 and 2015 are shown as black and blue lines in Fig. 3, respectively. As conditional distribution f(*p*|*R*), we chose the multivariate normal distribution.

$$ \mathrm{f}\left(p|R\right)\propto \exp .\left(-{\left({p}_{\mathrm{mod}}-{p}_{\mathrm{obs}}\right)}^{\mathrm{t}}\ {\Sigma}^{-1}\ \left({p}_{\mathrm{mod}}-{p}_{\mathrm{obs}}\right)/2\right) $$

where *p*_mod_ = *p*_mod_(*R*) is the solution of the PDE for a given *R*. The conditional distribution f(*p*|*R*) assesses the differences between the modeled *p*_mod_ and observed prevalences *p*_obs_. The covariance matrix Σ is estimated by following diagonal matrix:

$$ \Sigma =\operatorname{diag}\left({p}_{\mathrm{j}}\ \left(1-{p}_{\mathrm{j}}\right)/{n}_{\mathrm{j}}\right) $$

with age-specific prevalences *p*_j_ and the corresponding number of people *n*_j_ in the age group *j*. Choosing the covariance matrix as a diagonal matrix makes the implicit assumption that the prevalences *p*_j_ are stochastically independent. A justification for this assumption is the fact that people belonging to one age group are different from the people in another age group.

In a sensitivity analysis, we released the assumption of weakly informative priors (*R*(50) ~ U(2; 9), *R*(90) ~ U(1; 2)) and examined the impact on the estimation of R(50) and R(90). For this, we choose *R*(50) and *R*(90) from a bivariate normal distribution with mean (5.5, 1.5), standard deviation of 1 and 0.1 in *R*(50) and *R*(90), respectively, and a correlation coefficient of 0.9 between *R*(50) and *R*(90). These assumptions lead to the following covariance matrix for the joint distribution of *R*(50) and *R*(90):

$$ \left(\begin{array}{cc}{1}^2& 0.9\times 1\times 0.1\\ {}0.9\times 1\times 0.1& {0.1}^2\end{array}\right) $$

## Results

### Illness-death model

The age-specific prevalence *p*(*t*, *a*) = *I*(*t*, *a*)/{*H*(*t*, *a*) + *I*(*t*, *a*)} i.e., the percentage of people aged *a* at time *t* who are chronically ill, is the solution of the following partial differential equation (PDE):

3 $$ \left({\partial}_t+{\partial}_a\right)p=\left(1-p\right)\left\{i-p\left({m}_1-{m}_0\right)\right\} $$

In Eq. (3), ∂_t_ and ∂_a_ denote the partial derivatives with respect to *t* and *a*, respectively. The mathematical proof for Eq. (1) can be obtained from examining the change rates of the number of healthy and ill people in the illness-death model (*H* and *I* in Fig. 1) [17] or by using the theory of stochastic processes [2].

Eq. (3) implies that the excess mortality Δ*m* = *m*_1_ – *m*_0_ can directly be estimated from the incidence rate *i*, prevalence *p* and the temporal change of the prevalence ((∂_t_ + ∂_a_)*p*):

4 $$ \triangle m=\left[i-\frac{\left({\partial}_t+{\partial}_a\right)p}{1-p}\right]/p $$

Note that for direct estimation of the excess mortality Δ*m* by Eq. (2) only the incidence rate *i* and the prevalence based figures *p* and (∂_t_ + ∂_a_) *p* are necessary. No additional data are needed.

Instead of using Eq. (3) for a relation between the incidence, prevalence and mortality, an alternative way is possible by considering the prevalence-odds *θ*(*t*, *a*) = *I*(*t*, *a*)/*H*(*t*, *a*). For the prevalence-odds *θ* we find the following PDE, which is equivalent to Eq. (3):

5 $$ \left({\partial}_t+{\partial}_a\right)\theta =i-\theta \left({m}_1-{m}_0-i\right) $$

Equation (5) was first published by Brunet and Struchiner [18]. The derivation is given in an additional file [Additional file 1]. Compared to Eq. (3) the PDE (5) has the advantage of being linear. Solving PDEs like Eq. (3) and (5) is usually accomplished by transformation into an equivalent ordinary differential equation by the Method of Characteristics [15]. In case of Eq. (3), the resulting ordinary differential equation is of Ricatti type [19], which in general can only be solved numerically because an explicit representation of the general solution does not exist [20]. In case of the equivalent Eq. (5), however, an explicit representation of the solution indeed is possible. As detailed in the additional file [see Additional file 1] it holds:

6 $$ \theta \left(t,a\right)=\underset{0}{\overset{a}{\int }}i\left(t-s,a-s\right)\exp \left(-{\varphi}_{t,a}(s)\right) ds $$

For brevity, in Eq. (6) it was set ${\varphi}_{t,a}(x)={\int}_0^x\left[{m}_1-{m}_0-i\right]\left(t-x+\tau, a-x+\tau \right) d\tau$

The explicit representation of the solution *θ* in Eq. (6) allows to calculate *θ* with any prescribed accuracy, e.g. by Romberg integration [16], which we will use in the examples below. Applying the back-transformation *p* = *θ*/(1 + *θ*) yields the prevalence *p*.

For later use, we note that Eq. (3) can also be expressed in terms of the mortality rate ratio *R* and the general mortality *m* = *p m*_1_ + (1 – *p*) *m*_0_:

7 $$ \left({\partial}_t+{\partial}_a\right)p=\left(1-p\right)\left\{i-m\frac{p\left(R-1\right)}{1+p\left(R-1\right)}\right\} $$

### Direct estimation: dementia in the female population of Europe

**Fig. 4:**
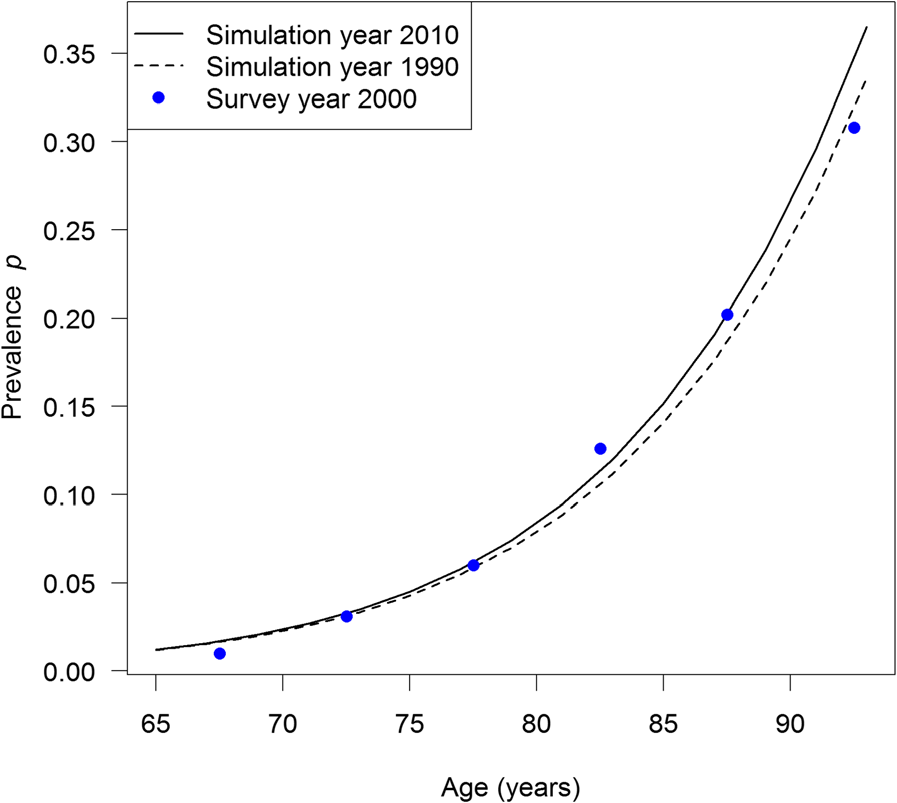
Simulated age-specific prevalence *p* of dementia in European women 1990 (solid black line) and 2010 (dashed black line). For comparison, the surveyed values in 2000 are plotted as blue dots

After calculating the prevalence-odds *θ* in years *t*_1_ and *t*_2_ by Eq. (6), the associated prevalences *p* = *θ*/(1 + *θ*) are calculated. Figure 4 shows the age-specific prevalences for the years *t*_1_ = 1990 (dashed line) and *t*_2_ = 2010 (solid line). To demonstrate that our simulated prevalence has a reasonable range, we additionally plotted the surveyed values for European women reported in [8]. The proposed method to estimate the excess mortality Δ*m* in the year *t* = 2000 is the direct application of Eq. (4). The partial derivative (∂_t_ + ∂_a_)*p* in Eq. (4) is approximated by a finite difference:

$$ \left({\partial}_t+{\partial}_a\right)p\left(t,a\right)\approx p\left(t+\triangle T/2,a+\triangle T/2\right)-p\left(t-\triangle T/2,a-\triangle T/2\right)/\triangle T $$

Then, the excess mortality Δ*m* can be estimated by plugging these numbers into Eq. (4). In case the mortality rate *m*_0_ of the non-diseased is known, the age-specific mortality rate ratio can be calculated by *R* = 1 + Δ*m*/*m*_0_. Table 1 shows the true and estimated values for *R* at different ages and various choices of Δ*T*.

**Table 1:** True and estimated mortality rate ratios

Age	True *R*	*ΔT* = 0.1		*ΔT* = 0.5		*ΔT* = 1		*ΔT* = 3		*ΔT* = 5		*ΔT* = 10	
		Est. *R*	rel. Err (%)	Est. *R*	rel. Err (%)	Est. *R*	rel. Err (%)	Est. *R*	rel. Err (%)	Est. *R*	rel. Err. (%)	Est. *R*	rel. Err. (%)
65	2.717	2.717	0.01	2.716	−0.06	2.708	−0.32	2.635	−3.0	2.495	−8.2	1.942	− 29
70	2.461	2.461	0.02	2.460	−0.04	2.455	−0.23	2.406	−2.2	2.312	−6.0	1.938	−21
75	2.229	2.229	0.00	2.228	−0.03	2.225	−0.18	2.191	−1.7	2.128	−4.6	1.868	−16
80	2.019	2.019	0.00	2.018	−0.02	2.016	−0.14	1.993	−1.3	1.948	−3.5	1.765	−12
85	1.829	1.829	0.00	1.828	−0.01	1.827	−0.11	1.810	−1.0	1.779	−2.7	1.647	−9.9
90	1.656	1.656	0.02	1.666	0	1.655	−0.07	1.643	−0.76	1.621	−2.1	1.528	−7.7
95	1.500	1.501	0.10	1.502	0.1	1.503	0.18	1.505	0.36	1.511	0.76	1.492	−0.54

Table legend: The estimated mortality rate ratios (Est. *R*) at different temporal distances between the two cross-sectional studies (Δ*T*) and different ages (first column) are compared to the true mortality rate ratios (True *R*, second column). The difference between the estimated and the true mortality rate ratio are given in terms of the relative error (rel. Err., in %)

From Table 1 we can see that the absolute relative Error increases as the temporal difference Δ*T* between the cross-sections increases and that absolute relative error increases as the age decreases. In the extreme case (age 60, Δ*T* = 10), the absolute relative error reaches nearly 30%. This indicates that in case two cross-sectional studies are separated by more three years (i.e., Δ*T* > 3) the method yields feasible results only in the higher age groups.

### Bayesian estimation of excess mortality in male diabetics from Germany

**Fig. 5:**
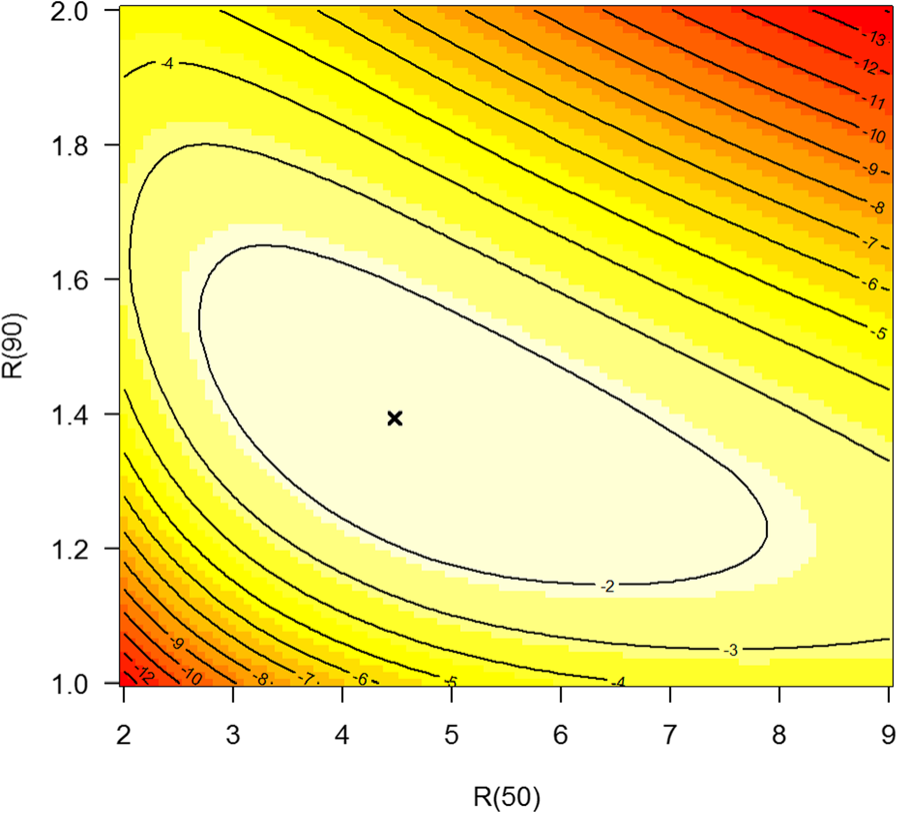
Contour plot of the posteriori likelihood of the mortality rate ratio *R* at ages 50 (abscissa) and 90 (ordinate). The maximum a posteriori (MAP) estimator is indicated as a black cross

The log-likelihood of the a-posteriori distribution f(*R*|*p*) ∝ f(*p*|*R*) × f(*R*) is shown in Fig. 5. The black cross indicates the maximum aposteriori (MAP) estimator for these data, which is given by *R*_MAP_(50) = 4.47 and *R*_MAP_(90) = 1.39. We obtain the estimates for *R*(50) and *R*(90) including 95% credibility intervals as shown in Table 2.

**Table 2:** Estimated mortality rate ratios for the diabetes data

Age	Mortaltiy rate ratio *R*	95% credibility interval	
50	4.47	4.17	4.78
90	1.39	1.33	1.46

Table legend: The estimated mortality rate ratios (*R*) at ages 50 and 90 with 95% credibility intervals

These values agree well with the empirical findings from the Danish Diabetes Register [14], where values slightly below 4 and slightly above 1.5 have been found for ages 50 and 90 years, respectively.

In the sensitivity analysis with bivariate normal prior distributions, the MAP estimator changed only slightly to *R*_MAP_(50) = 4.54 and *R*_MAP_(90) = 1.38.

## Discussion

In this work, we have described how the illness-death model can be used to obtain information about excess mortality in case prevalence and incidence are given. It turns out that the excess mortality can be calculated by the incidence rate, the prevalence and the temporal change of the prevalence (see Eq. (4)). In data where these figures are estimable, insights into the excess mortality of people with chronic diseases compared to the people without the disease can be gained.

As applications, simulated data about dementia and claims data about diabetes have been analyzed. For the dementia example we estimated the excess mortality directly and for the diabetes data we formulated a Bayesian approach. Both methods were based on aggregated data only (age-specific prevalence and incidence rate) and do not require data from individual subjects. Aggregated data can be found frequently in the literature, which makes the proposed method suitable for many applications, especially when the research question is aimed at population-wide measures. Here, we have chosen aggregated data about diabetes from the statutory health insurance in Germany based on about 35 million men. Based on the age-specific prevalence in 2009, we used non-informative priors for mortality rate ratio *R* and the PDE (7) to estimate the aposteriori likelihood of *R* given the age-specific prevalence in 2015. In this way, the PDE can therefore be seen as the data generating process underlying the prevalence data. In a sensitivity analysis, we used more informative prior distributions (bivariate normal) and found that the estimated values for the mortality rate ratios changed only slightly. Main reason for this robustness is due to the large number of people in the prevalence data.

Our approach has two limitations. The first limitation stems from the fact that Eqs. (3) and (5) are only valid if migration into and or from the considered population does not take place or if the prevalence of the chronic condition in migrants is similar to the prevalence in the resident population [21]. If migration happens on a considerable magnitude and if the prevalence in the migrants is substantially different from the residents, adoptions to Eq. (3) are possible [21]. The second limitation of our novel approach becomes visible in the simulation study about dementia: The two (or more) cross-sectional surveys for estimating the change of the prevalence should not be separated too much. In our simulation, the surveys should be conducted within a period of three years (or less) (i.e., Δ*T* ≤ 3) to have a relative error below 5%. If the two cross-sections are separated by ten years (Δ*T* = 10), the relative error has reached up to 30%. In the diabetes example, the two cross-sections were separated by six years (Δ*T* = 6). Based on this, we expect the relative errors of our estimates *R*(50) and *R*(90) to be about 10%. For comparison, the width of the credibility intervals for our estimates *R*(50) and *R*(90) have a similar magnitude. Thus, we would conclude a relative error of 10% in the mortality rate ratio is a rough estimate of the magnitude of accuracy that can be obtained from our method applied to these data.

In the current analysis, no attempt has been taken to examine the effect of smaller population sizes, i.e., how sampling uncertainty in the age-specific prevalence and incidence affects the estimates of the excess mortality. Furthermore, we have not analyzed the robustness of the estimation methods against misclassification error (i.e., false positive and false negative rates in input prevalence and incidence data). Questions about sample sizes and misclassification are currently analyzed and will be subject to a future paper providing more technical details.

## Conclusion

The described approach is useful to estimate the excess mortality of a chronic condition from aggregated incidence and prevalence data. The feasibility has been demonstrated in a simulation study about dementia and in claims data about diabetes in German men.

## Additional files

Additional file 1: Microsoft Word file (doc) providing details of the mathematical background of Eqs. (5) and (6). (DOC 34 kb)

Additional file 2: Script (plain text file, accessible via any text editor, e.g., Notepad, GNU Emacs etc) for the dementia simulation study, intended to use with the statistical software R (The R Foundation of Statistical Software). (R 4 kb)

## Data Availability

The source code for generating the data about dementia in Europe is available as an electronic supplement to this published article [Additional file 2]. The data for the diabetes example were taken from a published source [12], which has been cited in the text.

## Abbreviations

MAP: Maximum aposteriori
NHIS: National Health Interview Survey
PDE: Partial differential equation
