# Supplementary material for: New ways of estimating excess mortality of chronic diseases from aggregated data: insights from the illness-death model
Source: BMC Public Health. 2019 Jun 28;19:844. doi: 10.1186/s12889-019-7201-7 (PMC6599235; doi:10.1186/s12889-019-7201-7)
Supplement: Supplementary file 1 — Microsoft Word file (doc) providing details of the mathematical background of Eqs. (5) and (6). (DOC 34 kb) [file 12889_2019_7201_MOESM1_ESM.doc]

# Mathematical background to *New ways of estimating excess mortality of chronic diseases: Insights from the illness-death model* by *Ralph Brinks, Thaddäus Tönnies, Annika Hoyer*

## Derivation of Equation (5) of the main text

We write ∂*_t_ = ∂ /* ∂*t* and ∂*_a_ = ∂ /* ∂*a* for the partial derivative with respect to *t* and *a,* respectively. Let *H = H*(*t, a*) and *I = I*(*t, a*) denote the numbers of healthy and ill subjects, respectively (see Figure 1 in the main text). Then, the changes of *H* and *I* according to transition rates in the illness-death model are given by the following system of partial differential equations:

(∂*_t_* + ∂*_a_*) *H* = – [*i* + *m*_0_] *H,*  (A.1)

(∂*_t_* + ∂*_a_*) *I*  = *i* *H* –*m*_1_ *I.* (A.2)

Applying the quotient rule to the derivative of the prevalence-odds yields

(∂*_t_* + ∂*_a_*) *θ* = (∂*_t_* + ∂*_a_*) {*I*/*H*} = {*H* (∂*_t_* + ∂*_a_*) *I – I* (∂*_t_* + ∂*_a_*) *H*}*/H*²

= {(∂*_t_* + ∂*_a_*) *I – θ* (∂*_t_* + ∂*_a_*) *H*}/*H.*

Inserting Eqs. (A.1) and (A.2) yields Eq. (5) of the main text.

## Derivation of Equation (6) of the main text

Using the initial condition *H*(*t,* 0) *= H*_0_(*t*) for all *t* (i.e., *H*_0_ is the number of healthy newborns), the solution *H*(*t*, *a*) of (A.1) can be written as

$H(t,a)=H_{0}(t-a) \exp\left(-\int_{0}^{a} \left[m_{0}(t-a+s,s)+i(t-a+s,s)\right] ds\right)$.

If we consider only diseases contracted after birth, i.e. *I*(*t*, 0) = 0 for all *t*, the PDE (A.2) has the solution *I*(*t*, *a*) given by

$$I(t,a)=\int_{0}^{a} i(t-s,a-s) H(t-s,a-s) \exp\left(-\int_{0}^{s} m_{1}(t-a+\varsigma,\varsigma) d\varsigma\right) ds.$$

Inserting these equations for *H* and *I* into the definition of the prevalence-odds *θ* = *I*/*H* yields Eq. (6) of the main text.
